# Evaluation of the mobility of heavy metals in the sediments originating from the post-galvanic wastewater treatment processes

**DOI:** 10.1007/s10653-023-01686-6

**Published:** 2023-07-26

**Authors:** Paweł Lejwoda, Henryk Świnder, Maciej Thomas

**Affiliations:** 1 Department of Environmental Monitoring, Central Mining Institute in Katowice, Plac Gwarków 1, 40-166 Katowice, Poland; 2 Faculty of Environmental Engineering and Energy, Cracow University of Technology, Warszawska 24, 31-155 Kraków, Poland

**Keywords:** Mobility, Heavy metals, Galvanic wastewater, Sediments, Electroplating, Galvanic industry

## Abstract

The article presents the assessment of heavy metals mobility in sediments from the process of galvanic wastewater treatment (pH 2.5, Co 1.5 mg/L, Cr^6+^  < 0.02 mg/L, Cr_(total)_ 62 mg/L, Cu 110 mg/L, Ni 129 mg/L and Pb 59 mg/L) based on the use of hydroxides (Ca(OH)_2_, NaOH) as well as inorganic and organic sulphur compounds (Na_2_S, sodium dimethyldithiocarbamate (DMDTC), sodium trithiocarbonate (Na_2_CS_3_), trimercapto-s-triazine trisodium salt, TMT). The leachability was assessed after 1, 7, 14 and 21 days of sediment contact with the leaching agent (deionized water). FeCl_3_ was used as a coagulant. The efficiency of metal removal changed within a range of 99.67–99.94% (for NaOH), 98.80–99.75% (for TMT), 99.67–99.92% (for DMDTC), 99.67–99.91 (for Na_2_CS_3_). The heavy metal content in the obtained precipitates changed within the following ranges: 0.1–0.2 g/kg (Co), 9.8–14.7 g/kg (Cr), 23.6–39.8 g/kg (Cu) 30.5–43.2 g/kg (Ni), 24.3–33.1 g/kg (Pb) and 12.2–18.7 g/kg (Cd). The leachability tests revealed the release of 34–37% of Cd, 6.4–7.5% of Ni and 0.06–0.07% of Cu after using an excess of Na_2_CS_3_ as the precipitant. The use of NaOH resulted in the release of 0.42–0.46% of Cr from the sediment, and the use of TMT 0.03–0.34% of Ni. The best immobilization of heavy metals was observed in the case of the precipitate resulting from the use of DMDTC as a precipitating agent. The findings may be useful for predicting the mobility of heavy metals in the sludge and assessing the risk involved so as to support their removal and management.

## Introduction

The process of corrosion negatively affects objects, structures and materials, depriving them of aesthetic values and functional parameters. In extreme cases, it leads to their complete destruction. Every year in the USA, corrosion processes generate losses of up to hundreds of billions of dollars (Koch et al., 2005). To prevent these phenomena, many effective protection methods are applied, including the use of anti-corrosion paints and anodic or cathode protection (Vijayan & Al-Maadeed, 2019). The electroplating industry offers to coat the surface of a material susceptible to corrosion with another metal which is resistant to this phenomenon. The surfaces protected in this way are characterized by tightness, durability and aesthetic values, such as gloss, colour etc., which are advantageous from the point of view of utility (Loftis & Abdel-Fattah, 2019). The galvanization process involves using galvanic baths containing e.g. metal salts (NiSO_4_, NiCl_2_, CuSO_4_, CrCl_3_ etc.), which create protective layers on the materials subjected to treatment as a result of current-free processes (chemical reactions) or electrolytic processes (due to electric current action). Galvanic baths, apart from heavy metals, contain substances that improve the efficiency of the electroplating process and the quality of the resulting coatings, e.g. buffer compounds, brightening agents, carrying agents, surfactants and many others (Benvenuti et al., 2014). In the event of a decrease in the electroplating process efficiency, the galvanic baths are regenerated or replaced with new ones, and the process solutions become waste containing significant amounts of heavy metals, such as Cr, Cu, Ni, Cd, Co, Pb, Zn, etc. The galvanic bath electroplating and regeneration process generates wastewater, the composition and physicochemical properties of which depend on many factors. The chemical composition of wastewater depends, among others, on the type of protective coatings produced, which is related to the type of bath and the processes used for this purpose. Wastewater from the process of electrochemical nickel plating may contain 547.2 mg/L of nickel (Peng et al., 2020), 283 mg/L of copper from the copper plating process (Konstantinos et al., 2011), and 81 mg/L of zinc from the zinc plating process (Bian et al., 2022). Heavy metal cations are present in galvanic wastewater in various concentrations, e.g. Cu 59.0 mg/L, Cd 4.50 mg/L, Zn 22.7 mg/L (Thomas et al., 2021), Cu 70.8 mg/L, Sn 3.36 mg/L, Ni 1.1 mg/L (Thomas et al., 2018a, 2018b), Cu 49.5 mg/L (Thomas et al., 2018c), Sn 3100 mg/L (Thomas et al., 2017). Due to the presence of heavy metals, this wastewater has a toxic effect on the microorganisms in the active sludge and inhibits biological wastewater treatment processes (Głodniok et al., 2016). Before being discharged into the sewage system or natural environment, galvanic wastewater must be subjected to treatment. Wastewater treatment methods include among others membrane techniques, ion exchange (Sofińska-Chmiel & Kołodyńska, 2018) and, frequently, sorption methods where synthetic sorbents or sorbents of natural origin are used (Charazińska et al., 2021, 2022). However, the most commonly used are precipitation methods, which involve using reagents that react with heavy metal ions present in the wastewater. These reactions resulted in obtaining sparingly soluble sediments that contained heavy metals. A commonly used and economical method of removing heavy metals from galvanic wastewater is chemical precipitation with wastewater alkalizing compounds such as Ca(OH)_2_, NaOH, Mg(OH)_2_ (conventional treatment), which leads to the precipitation of sparingly soluble metal hydroxides (Blais et al., 2008). To improve the sedimentation properties of the sediment obtained as a result of metal precipitation reactions and to increase the efficiency of removing other pollutants contained in the treated wastewater, coagulants containing iron(II), iron(III) or aluminium compounds are added. Additionally, flocculants of different molecular weight and charge (cationic, anionic, non-ionic) are used to reduce the volume of the precipitates and to ensure their effective dewatering. In some cases, additional precipitants (other than hydroxides), such as Na_2_CS_3_ (Lejwoda et al., 2023; Thomas et al., 2021, 2023), Na_2_S (Lochyński et al., 2021), dimethylglyoxime (DMG) (Świnder & Lejwoda, 2021) must be used to increase the metal precipitation process efficiency. These processes generate significant amounts of hydrated sediment, which can be processed in order to recover and reuse the metals it contains (Brožová et al., 2021). Alternatively, the sediment is deposited in landfills, which, in the absence of adequate protection, creates a risk of uncontrolled heavy metals release into the environment. Effective treatment of industrial wastewater containing significant amounts of metal ions as well as protection of sludge against uncontrolled release of metal ions into the environment are important issues widely discussed and studied in the field of engineering and environmental protection. These studies should result in taking appropriate remedial measures so as to prevent the negative effects of toxic, carcinogenic and mutagenic substances on living organisms (Assi et al., 2016; Costa, 1997; Domingo, 1989; Genchi et al., 2020; Huff et al., 2007). The aim of the conducted research was to evaluate the mobility of heavy metals in sediments produced in the process of galvanic wastewater treatment by precipitation methods. Additionally, the influence of the type of the precipitating agent on the efficiency of precipitation of selected heavy metals (Co, Cr, Cu, Ni, Pb, Cd) from mixed galvanic wastewater as well as the degree of selected heavy metals immobilization were determined. For this purpose, aqueous extracts of the obtained precipitates were prepared by increasing contact times for the liquid and solid phases (1, 7, 14 and 21 days). In the investigations, the efficiency of precipitation and immobilization of heavy metals was compared after using four precipitants, i.e. calcium hydroxide (Ca(OH)_2_), sodium hydroxide (NaOH), sodium trithiocarbonate (Na_2_CS_3_), sodium dimethyldithiocarbamate (DMDTC) and trimercapto-s-triazine, trisodium salt (TMT).

## Material and methods

### Material

The researchers used wastewater from an electroplating plant in which the processes of chrome plating, copper plating, nickel plating and lead plating were applied. An average daily sample of wastewater was obtained by mixing unit samples that were collected every 1 h during the day in the amount of 1 L. The sample was transported and stored at 4℃ until the tests were performed. Selected physicochemical parameters of raw wastewater have been presented in Table 1.

**Table 1:** Selected physicochemical properties of raw galvanic wastewater

Parameter	Unit	Result ± measurement uncertainty
pH	–	2.5 ± 0.1
Colour	mg Pt/L	22.0 ± 4.4
Turbidity	NTU	210 ± 21
Chloride	mg/L	950 ± 95
Sulphate	mg/L	1300 ± 130
Chemical Oxygen Demand, COD_(Cr)_	mg O_2_/L	545 ± 82
Total Organic Carbon, TOC	mg/L	180 ± 27
N_NH4_	mg/L	15.0 ± 1.5
N_(total)_	mg/L	65 ± 10
P_(total)_	mg/L	7.5 ± 0.8
Co	mg/L	1.5 ± 0.2
Cr^6+^	mg/L	< 0.02
Cr_(total)_	mg/L	62 ± 6
Cu	mg/L	110 ± 11
Ni	mg/L	129 ± 13
Pb	mg/L	59 ± 6
Cd	mg/L	61 ± 6

Calcium hydroxide (Ca(OH)_2_, analytical grade, Chempur, Poland), sodium hydroxide (NaOH, analytical grade, Chempur, Poland), DMDTC (40% solution of sodium dimethyldithiocarbamate, technical grade, Chemische Fabrik Wocklum Gebr. Hertin GmbH & Co. KG, Balve, Germany), TMT (15% solution of trimercapto-s-triazine, trisodium salt, technical grade, Merck, Darmstadt, Germany), sodium trithiocarbonate (Na_2_CS_3_, 45% solution of sodium trithiocarbonate, technical grade, Chemiqua, Cracow, Poland), anionic flocculant (Furoflock CW 277, technical grade, Chemische Fabrik Wocklum GmbH & Co. KG, Germany) and ferric chloride (FeCl_3_, analytical grade, Chempur, Poland) were used to precipitate heavy metals (Lejwoda et al., 2023). In addition, concentrated nitric acid (HNO_3_), hydrochloric acid (HCl) and sulphuric acid (H_2_SO_4_) (spectral pure, Merck, Darmstadt, Germany) and deionized water (< 0.05 µS/cm, Direct-Q3 UV, Merck Millipore, Burlington, USA) were used in the investigations.

### Methods

The heavy metal removal experiments were carried out at temperature of 20 ± 1 °C. The precipitation of metals in 500 mL wastewater samples was carried out using a magnetic stirrer. The mixing speed in the metal precipitation step was 200 rpm and 50 rpm for 1 min. during the flocculation of the sludge. Removal of heavy metals was carried out by increasing the pH of the wastewater by adding a 15% Ca(OH)_2_ suspension to pH 7–7.5, and then by adding 15% NaOH to pH 9–9.5. In the next step, the selected precipitating agent (DMDTC, TMT, Na_2_CS_3_) was added to complete removal of heavy metals. The end of precipitating reagent addition was determined by a drop test. The drop test was performed by adding 0.025 mL of DMDTC, TMT or Na_2_CS_3_ to 0.5 mL of the filtered wastewater sample (syringe filter, hydrophilic PTFE, 0.45 µm) (Lejwoda et al., 2023).

The end of the addition of the precipitating agent was determined by a negative drop test (no precipitation or change in colour of the sample). A slight excess of precipitating agent (1–2%) was used in each of the experiments. When TMT, DMDTC and Na_2_CS_3_ solutions were used, the pH of the wastewater was adjusted by adding 10% H_2_SO_4_ to pH 9–9.5. The correction resulted from the alkaline properties of the solutions. After the precipitation step was completed, 2.0 mL of a 0.05% anionic flocculant solution was added to the mixtureAfter the sedimentation process (30 min), samples of the treated sewage were collected, filtered (membrane filter 0.45 µm, hydrophilic PTFE) and subjected to composition tests described in the Analytical Methods section. The sediments were dewatered using a filter cloth with a grammage of 140–160 g/m^2^, then dried in the air and in a desiccator at a temperature of 20 ± 1 °C; next, they were averaged and tested in order to determine their composition and mobility of heavy metals. Aqueous extracts were prepared by weighing dried and averaged sediment samples obtained at the stage of heavy metal precipitation, and by mixing with deionized water in a ratio of 10 g of sediment per 100 mL of water. 16 water extracts were prepared (4 types of sediments, 4 leaching periods). After each of the leaching periods, i.e.: 1, 7, 14, 21 days, the samples were filtered through a 0.45 µm membrane filter, and the content of heavy metals released from the sediments in the filtrates was determined after 1, 7, 14 and 21 days of contact.

### Analytical methods

The content of metals (Cd, Co, Cr, Cu, Ni, Pb) in wastewater and sludge was determined using ICP-OES according to standard EN ISO 11885:2009 (Optima 5300DV, Perkin Elmer, USA). Yttrium (Y) was used as internal standard. The sediments were digested with aqua regia (HCl: HNO_3_, 3:1, v/v). The determination of metals was performed with the level of uncertainty of 10%, 15%, 20%, depending on the element and its concentration in the tested solution, a coverage factor of 2 and a significance level of 95%, without taking into account the uncertainty related to sampling. Concentrations of metals (Cd, Co, Cr, Cu, Ni, Pb) in aqueous extracts were determined using ICP-MS according to standard EN ISO 17294-2:2016 (NexION 300S, Perkin Elmer, USA). The determination of metals was performed with the level of uncertainty of 15%, a coverage factor of 2 and a significance level of 95%, without taking into account the uncertainty related to sampling. Rhenium (Re) was used as internal standard. Certified Reference Material TMDA-70.2 solution was used as a quality control sample. In both measurement techniques (ICP-OES and ICP-MS), the standard solutions used met the requirements of standards ISO 17025 and ISO 17034.

The Inolab pH/ION/Cond 750 multiparameter (WTW, Germany) was used to measure the pH-value according to standard EN ISO 10523:2012 (accuracy of ± 0.1 pH). The colour and turbidity of the wastewater were determined using a PF-11 spectrophotometer (Macherey–Nagel, Germany), (uncertainty of 20% and 10%). The COD-value was determined by the miniaturized dichromate method using a PF-11 spectrophotometer (Macherey–Nagel, Germany) (uncertainty of 15%). TOC was determined using Nanocolor® TOC 60 test kits, with endpoint detection based on the use of a PF-11 spectrophotometer (Macherey–Nagel, Germany), (uncertainty of 15%). Total organic carbon determination was performed in two stages. In the first step of TOC determination, inorganic carbon was removed from the sample by using NaHSO_4_ and vigorously mixing the sample (500 rpm, 10 min). In the second stage, decomposition of organic compounds (Na_2_S_2_O_8_, 120 °C, 120 min) was carried out, followed by spectrophotometric measurement (λ = 585 nm) of the change in absorbance of the sodium salt solution (thymol blue, sodium salt solution), caused by a release of CO_2_. Determination of N_(total)_ was conducted by the two-stage spectrophotometric method with the use of Nanocolor®Total Nitrogen 220 (uncertainty of 15%). In the first stage, mineralization of the wastewater sample (Na_2_S_2_O_8_ + H_2_SO_4_, 120 °C, 30 min) was performed, and in the second stage—determination of nitrogen compounds after their reaction with 2,6-dimethylphenol in a mixture of H_2_SO_4_ and H_3_PO_4_.

Mohr’s method was used to determine chloride according to standard ISO 9297:1994 (uncertainty of 10%) and the concentration of sulfate was determined by gravimetric method according to standard ISO 9280:2002 (uncertainty of 10%). Nessler's method was used to determine N_NH4_ according to standard ISO 7150–1:1984 (uncertainty of 10%) and for determination of P_(total)_ the method described in standard ISO 18412:2005 was used, (uncertainty of 10%). Uncertainty of testing—expanded, k = 2, confidence level 95%.

## Results and discussion

The results of investigations into raw and treated wastewater using the conventional treatment (Ca(OH)_2_ + NaOH) as well as TMT, DMDTC and Na_2_CS_3_ have been given in Table 2

**Table 2:** Chemical composition of wastewater before and after treatment process

Parameter	Unit	Raw wastewater	Sample no. 1 Ca(OH)_2_ + NaOH conventional treatment	Sample no. 2 Ca(OH)_2_ + NaOH + TMT	Sample no. 3 Ca(OH)_2_ + NaOH + DMDTC	Sample no. 4 Ca(OH)_2_ + NaOH + Na_2_CS_3_
pH	–	2.5 ± 0.1	9.3 ± 0.1	9.2 ± 0.1	9.4 ± 0.1	9.3 ± 0.1
Colour	mg Pt/L	22.0 ± 4.4	< 10	< 10	< 10	< 10
Turbidity	NTU	210 ± 21	< 10	< 10	< 10	< 10
Chloride	mg/L	950 ± 95	1500 ± 150	1620 ± 162	1600 ± 160	1690 ± 169
Sulphate	mg/L	1300 ± 130	1250 ± 125	1220 ± 122	1290 ± 129	1280 ± 128
Chemical Oxygen Demand, COD_(Cr)_	mg O_2_/L	545 ± 82	320 ± 48	480 ± 72	325 ± 49	330 ± 50
TOC	mg/L	180 ± 27	90 ± 14	120 ± 18	85 ± 13	75 ± 11
N_NH4_	mg/L	15.0 ± 1.5	10 ± 1	11.0 ± 1.1	9.0 ± 0.9	11.0 ± 1.1
N_(total)_	mg/L	65 ± 10	36 ± 5	55 ± 8	40 ± 6	35 ± 5
P_(total)_	mg/L	7.5 ± 0.8	0.50 ± 0.05	0.20 ± 0.02	0.40 ± 0.04	0.50 ± 0.05
Co	mg/L	1.5 ± 0.2	0.005 ± 0.001	0.005 ± 0.001	0.005 ± 0.001	0.005 ± 0.001
Cr^6+^	mg/L	< 0.02	< 0.02	< 0.02	< 0.02	< 0.02
Cr_(total)_	mg/L	62 ± 6	0.04 ± 0.01	0.22 ± 0.04	0.05 ± 0.01	0.06 ± 0.01
Cu	mg/L	110 ± 11	0.10 ± 0.02	0.85 ± 0.11	0.09 ± 0.02	0.12 ± 0.02
Ni	mg/L	129 ± 13	0.10 ± 0.02	1.55 ± 0.31	0.21 ± 0.04	0.11 ± 0.02
Pb	mg/L	59 ± 6	0.04 ± 0.01	0.15 ± 0.03	0.08 ± 0.02	0.08 ± 0.02
Cd	mg/L	61 ± 6	0.06 ± 0.01	0.17 ± 0.03	0.11 ± 0.02	0.10 ± 0.02

The conducted research has revealed that the wastewater from galvanic processes used in the investigations was characterized by various concentrations of heavy metals. The highest metal concentrations were recorded in the case of copper (110 mg/L) and nickel (129 mg/L), followed by total chromium (62 mg/L) and lead (59.6 mg/L).

The studies of other authors indicate a significant variation in the composition of galvanic wastewater in terms of heavy metal content. The concentration and type of heavy metals contained in raw wastewater depend mainly on *(i)* types of processes used in the electroplating plant (copper plating, chrome plating, lead plating, galvanizing, etc.), *(ii*) the share of individual processes in the plant's output, *(iii)* the applied methods of galvanized components rinsing (jet rinsing, dip rinsing, etc.), *(v)* scrubbers used (recovery, cascade, permanent scrubbers with periodic water changes, etc.), *(vi*) total water consumption, *(vii)* degree of automation and *(viii)* general technical and technological level of the plant. Studies by other authors indicate that in the case of the wastewater from the chrome plating process (pH 4), the concentration of Cu, Cr, Ni, and Pb may reach 0.11 mg/L, 3.38 mg/L, 7.53 mg/L and 1.19 mg/L, respectively. The concentrations of Cu, Cr, Ni and Pb in the wastewater from electrochemical processes, where cyanide baths (pH 4) are used, can reach 5.20 mg/L, 2.11 mg/L, 35.56 mg/L and 0.01 mg/L, respectively. Typically rinse waters contain smaller amounts of heavy metals, e.g. 0.62 mg/L of Cu, 0.24 mg/L of Cr, 2.97 mg/L of Ni and 0.03 mg/L of Pb (Rahman et al., 2016). On the other hand, the wastewater from the process of printed circuit boards chemical and electrochemical treatment contains different amounts of copper, depending on the type of process from which it is derived, i.e., 3–20 mg/L (alkaline and acid etching processes), 0.1–0.5 mg/L (chemical copper plating), 0.5–3.0 mg/L (electrolytic copper plating) and 10–60 mg/L (brushing) (Thomas et al., 2014). On the other hand, the wastewater from the process of copper wires electrochemical tinning contained 3100 mg/L of Sn, 27.6 mg/L of Fe, 2.41 mg/L of Ni and 1.46 mg/L of Pb (Thomas et al., 2017). Additionally, the conducted research revealed that the tested wastewater did not contain chromium(VI), which may indicate the use of highly effective processes of reducing chromium(VI) to chromium(III) in the plant. The applied wastewater treatment processes enabled obtaining the treated wastewater characterized by an alkaline reaction of pH > 9, colour and turbidity of < 10 mg Pt/L and < 10 NTU, respectively. Due to the use of iron(III) chloride as a coagulant, the concentration of chlorides in the treated wastewater increased from 950 mg/L to even 1690 mg/L in the case where Na_2_CS_3_ was used as the precipitation reagent. In all cases, the cobalt concentration decreased from 1.5 mg/L to 0.005 mg/L, regardless of the precipitating agent applied. Total chromium, copper, nickel and lead concentrations varied in the ranges of 0.04–0.22 mg/L, 0.09–0.85 mg/L, 0.1–1.55 mg/L and 0.04–0.15 mg/L, respectively. The efficiency (%) of selected metals precipitation from the tested wastewater is presented in Tab. 3.

**Table 3:** The effectiveness of removal of Co, Cr, Cu, Ni, and Pb from wastewater

Parameter	Unit	Sample no. 1 Ca(OH)_2_ + NaOH conventional treatment	Sample no. 2 Ca(OH)_2_ + NaOH + TMT	Sample no. 3 Ca(OH)_2_ + NaOH + DMDTC	Sample no. 4 Ca(OH)_2_ + NaOH + Na_2_CS_3_
Co	%	99.67	99.67	99.67	99.67
Cr_(total)_	%	99.94	99.65	99.92	99.90
Cu	%	99.91	99.23	99.92	99.89
Ni	%	99.92	98.80	99.84	99.91
Pb	%	99.93	99.75	99.86	99.86
Cd	%	99.90	99.72	99.82	99.83

The use of selected precipitating agents (Ca(OH)_2_, NaOH, TMT, DMDTC and Na_2_CS_3_) resulted in the precipitation of 96.56–99.94% of metals contained in the tested wastewater. The applied combinations of precipitants were characterized by comparable efficiency. In the case of conventional treatment, the use of Ca(OH)_2_ and NaOH resulted in the precipitation of heavy metals in the form of hydrated metal hydroxides according to the general reaction equations:

1 $${\text{Me}}^{{{2} + }} + {\text{ Ca}}\left( {{\text{OH}}} \right)_{{2}} \to {\text{ Me}}\left( {{\text{OH}}} \right)_{{2}} \downarrow \, + {\text{ Ca}}^{{{2} + }}$$

2 $${\text{Me}}^{{{2} + }} + {\text{ 2NaOH }} \to {\text{ Me}}\left( {{\text{OH}}} \right)_{{2}} \downarrow \, + {\text{ 2Na}}^{ + }$$

As indicated in the literature (Lipiec & Szmal, 1996; Minczewski & Marczenko, 2022), the presence of cobalt ions leads to the precipitation of hydroxide salts and, next, cobalt hydroxide, cobalt(II) salts being easily oxidized with oxygen from the air to cobalt(III) salts (reactions: 3–5):

3 $${\text{Co}}^{{{2} + }} + {\text{ OH}}^{ - } + {\text{ Cl}}^{ - } \to {\text{ Co}}\left( {{\text{OH}}} \right){\text{Cl}}$$

4 $${\text{Co}}\left( {{\text{OH}}} \right){\text{Cl }} + {\text{ OH}}^{ - } \to {\text{ Co}}\left( {{\text{OH}}} \right)_{{2}} \downarrow \, + {\text{ Cl}}^{ - }$$

5 $${\text{4Co}}\left( {{\text{OH}}} \right)_{{2}} + {\text{ O}}_{{2}} + {\text{ 2H}}_{{2}} {\text{O }} \to {\text{ 4Co}}\left( {{\text{OH}}} \right)_{{3}} \downarrow$$

In the case of chromium(III), amphoteric Cr(OH)_3_ is precipitated, which may dissolve in NaOH used as precipitant (reactions: 6–7):

6 $${\text{Cr}}^{{{3} + }} + {\text{ 3OH}}^{ - } \to {\text{ Cr}}\left( {{\text{OH}}} \right)_{{3}} \downarrow$$

7 $${\text{Cr}}\left( {{\text{OH}}} \right)_{{3}} \downarrow \, + {\text{ OH}}^{ - } \to {\text{ Cr}}\left( {{\text{OH}}} \right)_{{4}}^{ - }$$

In the case of copper, nickel and lead cations, Cu(OH)_2_, Ni(OH)_2_, Pb(OH)_2_, respectively, are precipitated. The use of high pH values for lead precipitation can result in its digestion and conversion to soluble Pb(OH)_4_^2−^, according to the reaction (8) equation:

8 $${\text{Pb}}\left( {{\text{OH}}} \right)_{{2}} \downarrow \, + {\text{ 2OH}}^{ - } \to {\text{ Pb}}\left( {{\text{OH}}} \right)_{{4}}^{{{2} - }}$$

Exceeding the pH values that are suitable for the precipitation of a given hydroxide by using an increased amount of NaOH may therefore lead to secondary digestion of the precipitated hydroxides and increased concentration of a given metal in the wastewater. It is clear that all metals precipitate as hydroxides due to the addition of NaOH and Ca(OH)_2_ but the overall efficiency of the precipitation processes depends, among others, on the composition and physicochemical properties of wastewater, type of coagulant used, reaction time, etc. and has to be considered individually for the specific type of wastewater and precipitation process conditions. Nevertheless, some general principles regarding the process of heavy metals precipitation in the form of hydroxides can be formulated. For example, studies by other authors indicate that for each metal, it is possible to obtain a curve with a maximum point representing the greatest percentage efficiency of metal removal from the tested wastewater, which corresponds to the minimum hydroxide solubility. For example, for Pb and Cu these ranges are 7.8–8.8 and 8.1–11.1, respectively. In this work, a pH of 9.0–9.5 was applied, which was sufficient to effectively precipitate all the metals contained in the wastewater. The results are consistent with those reported in the literature (Eckenfelder, 2000; Hautala et al., 1977; Kim et al., 2002; Pang et al., 2009). Additionally, literature data indicates that the percentage efficiency of lead removal depends on the initial lead concentration in the wastewater; for 1.5, 3, 7 and 14 mg/L of Pb, the precipitation efficiency reached 69.0, 93.3, 96.9 and 98.3%, respectively (Pang et al., 2009). The data suggests that precipitation processes may be more efficient in the case of wastewater containing increased amounts of heavy metals (Ayoub et al., 2001; Daniels, 1975). Due to the fact that the investigated wastewater contained significant amounts of copper and nickel (110 and 129 mg/l, respectively), they could have contributed to the overall high efficiency of precipitation of all the metals in the wastewater. In addition, literature data indicates that in the case of amphoteric hydroxides (with an increased amount of hydroxide ions and after exceeding the minimum solubility point), their re-digestion accompanied by the formation of hydroxocomplexes may occur (Maruyama et al., 1975; Saari et al., 1998). This negative phenomenon was not observed in the case of the tested wastewater. If the wastewater contains complexing compounds, which hinder the efficient precipitation of heavy metals in the form of hydroxides, additional reagents such as TMT, DMDTC or Na_2_CS_3_ are used. In the case of the wastewater used in the research, no significant difference was found between the efficiency of metal precipitation using the above reagents and conventional treatment with Ca(OH)_2_ and NaOH. This could have been related to the low content of complexing compounds in the wastewater, which did not substantially affect the efficiency of metal cations precipitation with the use of hydroxides. It should be noted, however, that in the event TMT, DMDTC or Na_2_CS_3_ are used, the precipitate obtained is a mixture of metal hydroxides and corresponding dimethyldithiocarbamates, trithiocarbonates or trimercapto-s-triazine compounds.

For instance, in the case of Na_2_CS_3_ precipitated sludge will be a mixture of mainly sparingly soluble metal hydroxides, trithiocarbonates and sulfides, in accordance with following Eqs. (9–11):

9 $${\text{Me}}^{{{2} + }} + {\text{2OH}}^{ - } \, \to {\text{ Me}}\left( {{\text{OH}}} \right)_{{2}} \downarrow$$

10 $${\text{Me}}^{{{2} + }} + {\text{S}}^{{{2} - }} \, \to {\text{ MeS}} \downarrow$$

11 $${\text{Me}}^{{{2} + }} + {\text{CS}}_{{3}}^{{{2} - }} \, \to {\text{ MeCS}}_{{3}} \downarrow$$

The investigations of other authors depicted that the removal of chelated copper from wastewater by replacement precipitation by using ferrous compound was related to the molar ratio of Fe^2+^/Cu^2+^. When the mentioned ratio increased to 12, concentration of copper in wastewater decreased from 25 to 0.38 mg/L, while the Cu^2+^/EDTA ratio in wastewater was 1:1 (Jiang et al., 2010). For cadmium, the application of coprecipitation with 100 mg/L ferric chloride at pH 9 removed 97% of cadmium. On the other hand the application of alum (Al_2_(SO_4_)_3_) instead of ferric chloride at pH 9 removed only 91.5% of cadmium (El‐Gohary et al., 1979). Our findings correspond to the literature data. The chemical composition of the sediments (metal content) obtained by applying the above mentioned wastewater treatment methods has been presented in Table 4.

**Table 4:** Chemical composition of the sediments

Parameter	Unit	Sample no. 1 Ca(OH)_2_ + NaOH conventional treatment	Sample no. 2 Ca(OH)_2_ + NaOH + TMT	Sample no. 3 Ca(OH)_2_ + NaOH + DMDTC	Sample no. 4 Ca(OH)_2_ + NaOH + Na_2_CS_3_
Co	g/kg	0.20 ± 0.03	0.15 ± 0.02	0.10 ± 0.02	0.19 ± 0.03
Cr	g/kg	14.0 ± 2.1	13.9 ± 2.1	9.8 ± 1.5	14.7 ± 2.2
Cu	g/kg	31.6 ± 4.7	31.0 ± 4.7	23.6 ± 3.5	39.8 ± 6.0
Ni	g/kg	43.2 ± 6.5	41.3 ± 6.2	30.5 ± 4.6	42.5 ± 6.4
Pb	g/kg	31.3 ± 4.7	33.1 ± 5.0	24.3 ± 3.6	28.2 ± 4.2
Cd	g/kg	17.5 ± 2.6	17.1 ± 2.6	12.2 ± 1.8	18.7 ± 2.8

**Fig. 1:**
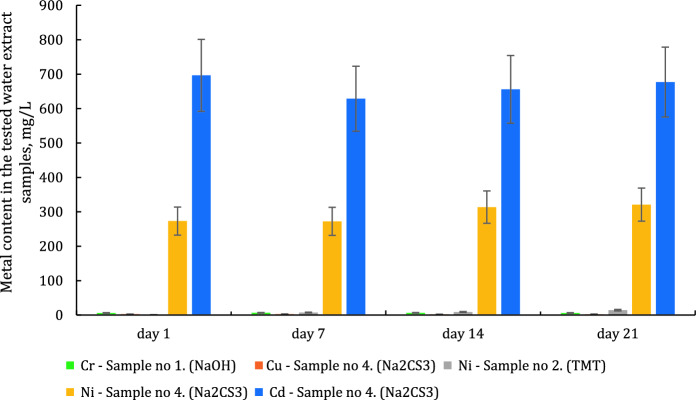
Metal content in the tested water extract samples (for the ratio of sediment mass to water volume reaching 1:10), according to PN-G-11010:1993 under static conditions, at a temp. of 20 ± 1 °C), measurement uncertainty ± 15%

**Fig. 2:**
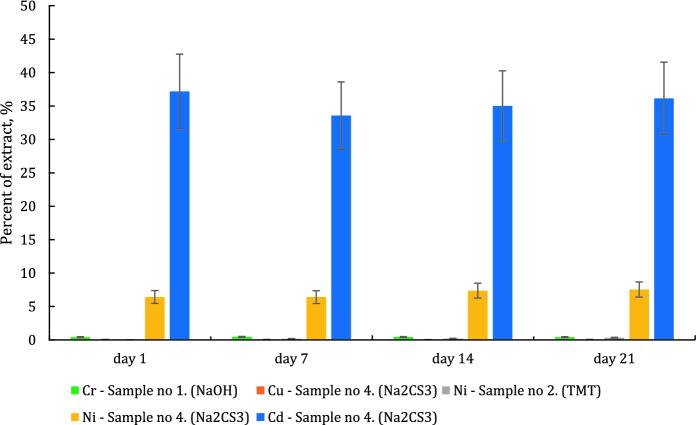
Metal content in the tested water extract samples (for the ratio of sediment mass to water volume reaching 1:10), according to PN-G-11010:1993 under static conditions, at a temp. of 20 ± 1 °C, measurement uncertainty ± 15%

In the subsequent stage of investigations, water extracts were prepared and the content of individual metals was determined after 1, 7, 14 and 21 days of the sediment contact with deionized water. The results of these tests are given in Table 5 and in Fig. 1. Additionally, Fig. 2 shows the % content of selected metals leached under test conditions.

**Table 5:** Metal content in the tested water extract samples (for the ratio of sediment mass to water volume reaching 1:10, according to PN-G-11010:1993 under static conditions, at a temp. of 20 ± 1 °C)

Sample	Parameter	Unit	After 1 day	After 7 days	After 14 days	After 21 days
Sample no. 1 Ca(OH)_2_ + NaOH conventional treatment	Cr	mg/L	5.87 ± 0.88	6.44 ± 0.97	6.24 ± 0.94	5.92 ± 0.89
	Ni	mg/L	< 0.02	< 0.02	< 0.02	< 0.02
	Cd	mg/L	< 0.02	< 0.02	< 0.02	< 0.02
	Co	mg/L	< 0.02	< 0.02	< 0.02	< 0.02
	Pb	mg/L	< 0.02	< 0.02	< 0.02	< 0.02
	Cu	mg/L	< 0.02	< 0.02	< 0.02	< 0.02
Sample no. 2 Ca(OH)_2_ + NaOH + TMT	Cr	mg/L	< 0.02	< 0.02	< 0.02	< 0.02
	Ni	mg/L	1.05 ± 0.16	7.33 ± 1.10	8.80 ± 1.32	14.0 ± 2.1
	Cd	mg/L	< 0.02	< 0.02	< 0.02	< 0.02
	Co	mg/L	< 0.02	< 0.02	< 0.02	< 0.02
	Pb	mg/L	< 0.02	< 0.02	< 0.02	< 0.02
	Cu	mg/L	< 0.02	< 0.02	< 0.02	< 0.02
Sample no. 3 Ca(OH)_2_ + NaOH + DMDTC	Cr	mg/L	< 0.02	< 0.02	< 0.02	< 0.02
	Ni	mg/L	< 0.02	< 0.02	< 0.02	< 0.02
	Cd	mg/L	< 0.02	< 0.02	< 0.02	< 0.02
	Co	mg/L	< 0.02	< 0.02	< 0.02	< 0.02
	Pb	mg/L	< 0.02	< 0.02	< 0.02	< 0.02
	Cu	mg/L	< 0.02	< 0.02	< 0.02	< 0.02
Sample no. 4 Ca(OH)_2_ + NaOH + Na_2_CS_3_	Cr	mg/L	< 0.02	< 0.02	< 0.02	< 0.02
	Ni	mg/L	273 ± 41	272 ± 41	314 ± 47	321 ± 48
	Cd	mg/L	697 ± 104	629 ± 94	656 ± 98	677 ± 102
	Co	mg/L	< 0.02	< 0.02	< 0.02	< 0.02
	Pb	mg/L	< 0.02	< 0.02	< 0.02	< 0.02
	Cu	mg/L	2.67 ± 0.40	2.72 ± 0.41	2.25 ± 0.34	2.50 ± 0.38

Chemical analysis of the obtained water extracts has revealed that under the experiment conditions, heavy metals from the precipitates may be released to the aquatic environment (deionized water). In the case of the mixture of NaOH and Ca(OH)_2_ used as a precipitant, the concentration of chromium in the aqueous extracts varied in the range of 5.87–6.44 mg/L. In the case of the precipitate obtained after the application of NaOH and Ca(OH)_2_ in the first stage, followed by TMT, a gradual increase in nickel leaching over time (1.05 mg/L after 1 day, 14.04 mg/L after 21 days) was observed. Despite the high efficiency of metal removal from the wastewater, in the case of the sediment obtained after the application of NaOH and Ca(OH)_2_, followed by Na_2_CS_3_, it was found that the leachability of copper varied in the range of 2.25–2.72 mg/L. At the same time, significant leachability of nickel (272–321 mg/L) and cadmium (629–697 mg/L) was observed. After the application of NaOH and Ca(OH)_2_ followed by DMDTC, the concentrations of all tested metals were below 0.02 mg/L. Figure 2 shows the percentage content of the elements that were leached from the sediment sample, on dried mass basis.

The conducted analysis has revealed that the highest leaching was noted for the sediment from processes in which NaOH and Ca(OH)_2_, followed by Na_2_CS_3_, were used as precipitating reagents. In these cases, the leachability of cadmium varied in the range of 34–37%, and in the case of nickel—in the range of 6.4–7.5%, based on the dry weight of the sediment treated with deionized water for 1–21 days. The above relationships were observed only in the cases where Na_2_CS_3_ was used. This could be explained by the fact that a slight 1–2% excess of the precipitating reagent was used in the case of each reagent (except for conventional treatment). Literature data indicates that under certain conditions the use of some excess of Na_2_CS_3_ or increased pH of wastewater during the precipitation process can increase the solubility of complexes (or salts produced in the presence of other substances in the wastewater, e.g., NH_4_^+^) of CS_3_^2−^ ions with metals, e.g., [Cu(CS_3_)_n_]^n−^, [Ni(CS_3_)_n_]^n−^, [Cd(CS_3_)_n_]^n−^, KCuCS_3_, NH_4_CuCS_3_, Zn(NH_3_)_2_CS_3_ and others (Bobrowska-Krajewska et al., 1994; Gattow & Behrendt, 1977; Thomas et al., 2021). Therefore, the use of a slight excess of the precipitating reagent may lead to the formation of soluble complex compounds and secondary contamination of the sediment with heavy metal ions, which are easily released from it during the leaching efficiency test with deionized water. (Świerk et al., 2007) investigated of heavy metals leaching from galvanic sewage sludge from electroplating plant using various extracting solutions e.g. water. The results of studies showed that the amounts of copper, nickel and chromium released from the industrial sludge were from 0.07% even to 99% of their total contents. Similarly, (Ozgul & Sabriye, 2012) used three extraction tests, synthetic precipitation leaching procedure (SPLP), American Society of Testing and Material (ASTM) method and rain water leaching to investigate the possibility of extracting metals from galvanic sludge. The authors found that Cr, Ni, and Zn leached above the drinking water standards as well as this leaching results higher with rain water thus possibly posing a potential risk to groundwater with climate conditions. A similar problem applies to the sludge originated from WWTP and designed to the compositing process. Some researchers claimed (Janas et al., 2018) that metals in sewage sludge, which undergo various transformations, are very difficult to immobilize. The application of calcium oxide and other supporting compounds do not affect radically the increase of leaching of the analyzed elements from the sludge. In this case, alkaline calcium compounds may have a protective effect. In the case of sediments containing significant amounts of amphoteric metal hydroxides, this process may lead to their dissolution and leaching of metal cations due to the increased of pH value.

## Conclusions

Each of the applied methods of wastewater treatment allowed achieving a high efficiency of heavy metals removal. The content of metals in the wastewater treated with NaOH, Ca(OH)_2_ (conventional treatment) and NaOH, Ca(OH)_2_ used as precipitants, and, next, additionally with Na_2_CS_3_ or DMDTC, did not exceed 0.005 mg/L for cobalt, 0.06 mg/L for total chromium, 0.12 mg/L for copper, 0.21 mg/L for nickel and 0.08 mg/L for lead. The highest concentrations of metals in the treated wastewater were observed in the case of TMT used as a precipitating agent, i.e. cobalt—0.005 mg/L, total chromium—0.22 mg/L, copper—0.85 mg/L, nickel—1.55 mg/L and lead—0.15 mg/L. The leachability tests carried out for the obtained precipitates revealed the leaching of cadmium within a range of 34–37% in relation to the total content after the application of Na_2_CS_3_; 0.42–0.46% of chromium in relation to the total content after the application of NaOH and Ca(OH)_2_; 0.03–0.34% of nickel in relation to the total content after the application of Na_2_CS_3,_ and 6.4–7.5% of nickel after TMT treatment; 0.06–0.07% of copper in relation to total content after using Na_2_CS_3_. The investigations indicate that DMDTC is the best agent for immobilizing heavy metals in sediments. The concentrations of metals did not exceed the upper limit of quantification in any of the tested water extracts of the sediment produced after DMDTC application. In order to determine the safety of storing sediments with a high content of heavy metals under real conditions, the variability of the composition and physicochemical properties of natural waters should be taken into account.

## Data availability and materials

The datasets supporting the conclusions of this article are included within the article.

## Abbreviations

COD: Chemical oxygen demand
DMDTC: Sodium dimethyldithiocarbamate
DMG: Dimethylglyoxime
ICP-OES: Inductively coupled plasma optical emission spectrometry
ICP-MS: Inductively coupled plasma mass spectrometry
NTU: Nephelometric turbidity unit
pH: Logarithmic and inversely indicates the concentration of hydrogen ions in the solution
TMT: Trimercapto-s-triazine trisodium salt
TOC: Total organic carbon
WWTP: Wastewater treatment plants
